# Improving rolling bearing online fault diagnostic performance based on multi-dimensional characteristics

**DOI:** 10.1098/rsos.180066

**Published:** 2018-05-23

**Authors:** Chuanlei Yang, Hechun Wang, Zhanbin Gao, Xinjie Cui

**Affiliations:** College of Power and Energy Engineering, Harbin Engineering University, Harbin 150001, People's Republic of China

**Keywords:** rolling bearing, fault diagnosis, entropy, Holder coefficient, fractal box-counting dimension, grey relation algorithm

## Abstract

As the main cause of failure and damage to rotating machinery, rolling bearing failure can result in huge economic losses. As the rolling bearing vibration signal is nonlinear and has non-stationary characteristics, the health status information distributed in the rolling bearing vibration signal is complex. Using common time-domain or frequency-domain approaches cannot easily enable an accurate assessment of rolling bearing health. In this paper, a novel rolling bearing fault diagnostic method based on multi-dimensional characteristics was developed to meet the requirements for accurate diagnosis of different fault types and severities with real-time computational performance. First, a multi-dimensional feature extraction algorithm based on entropy characteristics, Holder coefficient characteristics and improved generalized fractal box-counting dimension characteristics was performed to extract the health status feature vectors from the bearing vibration signals. Second, a grey relation algorithm was employed to achieve bearing fault pattern recognition intelligently using the extracted multi-dimensional feature vector. This experimental study has illustrated that the proposed method can effectively recognize different fault types and severities after integration of the improved fractal box-counting dimension into the multi-dimensional characteristics, in comparison with existing pattern recognition methods.

## 1. Introduction

Rolling bearings are widely used in almost all types of rotating machinery [1]. Rolling bearing failure is one of the main causes of failure and damage to rotating machinery, and can result in huge economic losses [2–4]. Technology on rolling bearing fault diagnostics has become more and more advanced over the years and the demands on technology in industrial applications, training and education are increasing. In order to ensure reliable operation of the unit and reduce economic losses, it is necessary to develop a reliable and effective diagnostic method for rolling bearings. Among the many fault diagnostic methods for rolling bearings, vibration-based diagnostic methods have received much attention in the past few decades [5,6]. Bearing vibration signals contain a wealth of information about mechanical health status. This also makes it possible to extract the dominant features that characterize the state of mechanical health from vibration signals through signal processing techniques [7]. Currently, many signal processing techniques have been applied to the off-line fault diagnosis of bearings. However, owing to many nonlinear factors (e.g. stiffness, friction, clearance), bearing vibration signals (especially in a faulty condition) will exhibit nonlinear and unsteady characteristics [8]. In addition, the measured vibration signals contain not only information about the operating condition associated with the bearing itself, but also information about other rotating components in the machine [9]. Owing to the existence of large background noise, slight bearing fault information is easily submerged in the background noise and becomes difficult to extract. Therefore, conventional time-domain and frequency-domain methods do not easily achieve an accurate diagnosis of the health status of the bearing [10]. With the development of nonlinear dynamics, many nonlinear analytical techniques have been applied to identifying and predicting the complex dynamic nonlinearities of bearings [11]. Among them, the most typical one is to extract the fault signature frequency from the vibration signals through the combined usage of some advanced signal processing techniques (such as higher order spectra [12], wavelet package transform [13], Hilbert transform [14], empirical mode decomposition), and further evaluate the bearing health status by comparing the vibration signals with the theoretical characteristic frequency value. With the development of artificial intelligence [15], the procedure of bearing fault diagnosis has been gradually introduced into the field of signal pattern recognition. The validity and reliability are mainly decided by the selection of dominant eigenvectors that characterize the fault features [16]. Recently, some entropy-based methods (such as hierarchical entropy [17], fuzzy entropy [18], sample entropy [19], approximate entropy [20,21], hierarchical fuzzy entropy) have been proposed for extracting the dominant eigenvectors that characterize fault features from bearing vibration signals and have achieved some effect. After fault feature extraction, a pattern recognition technique is used to perform the diagnosis of bearing faults [16]. Nowadays, a variety of pattern recognition methods have been used in mechanical fault diagnosis, of which the most widely used are support vector machines (SVMs) [22] and artificial neural networks (ANNs) [23–25]. Among them, ANN training requires a large number of samples, especially samples with fault features. SVMs are based on statistical learning theory, are more easily generalized than ANNs and can ensure that local optimal solutions and global optimal solutions are consistent [26]. However, the accuracy of SVM classifiers depends on the selection of optimal parameters [26,27]. In order to ensure diagnostic accuracy, some optimization algorithms [28] are often used to improve the effectiveness of SVMs. With the aim of solving the problem that traditional time-domain and frequency-domain methods cannot easily make an accurate diagnosis of rolling bearings, a rolling bearing online fault diagnostic method is proposed based on multi-dimensional feature extraction theory and grey relation pattern recognition theory.

The rest of the paper is organized as follows. First, the methodology of the proposed approach is introduced in §2, and second the experimental study of the proposed approach is illustrated in §3. The conclusion is presented in §4.

## 2. Methodology

### 2.1. Multi-dimensional feature extraction

In this paper, a novel rolling bearing fault diagnostic method was developed to meet the requirements for accurate diagnosis of different fault types and different severities with real-time computational performance. First, a multi-dimensional feature extraction on the basis of entropy characteristics, Holder coefficient characteristics and improved generalized box-counting dimension characteristics was proposed for extracting health status feature vectors from the bearing vibration signals.

#### 2.1.1. Entropy characteristics

Entropy is a crucial concept in information theory and is a measure of information uncertainty and complexity of the signals [29]. Therefore, the information contained within the signals can be quantitatively described by entropy characteristics.

Suppose the bearing vibration signal is *f*. The signal is first sampled and discretized into a discrete signal sequence, i=1,2,…,n. Perform fast Fourier transform as follows:

2.1 $$F(k)=\sum_{i=0}^{n-1}\;f(i)\exp\left(-j\frac{2\pi }{n}\text{i}k\right)\;k=0,1,2,\ldots ,n-1.$$

After obtaining the signal spectrum, calculate the energy of each point,

2.2 $$s_{k}=|F(k){|}^{2}.$$

Calculate the total energy value of each point,

2.3 $$S=\sum_{k=0}^{n-1}s_{k}.$$

Calculate the ratio of the energy of each point to the total energy,

2.4 $$P_{k}=\frac{s_{k}}{S}=\frac{s_{k}}{\sum_{k=0}^{n-1}s_{k}}.$$

The Shannon entropy $E_{1}$ and exponential entropy $E_{2}$ can be defined as follows:

2.5 $$E_{1}=-\sum_{i=1}^{n}P_{i}\log_{e}P_{i}$$

and

2.6 $$E_{2}=\sum_{i=1}^{n}P_{i}{\text{e}}^{1-P_{i}}.$$

The entropy characteristics $\left[E_{1},E_{2}\right]$ are taken as one set of dominant feature vectors used for further rolling element bearing fault pattern recognition.

#### 2.1.2. Holder coefficient characteristics

The Holder coefficient algorithm evolves from the Holder inequality [30,31]. The Holder coefficient can be used to measure the degree of similarity of two signal sequences. It evolved from Holder inequality, and the definition of the Holder inequality is as follows.

For any vector $X={\left[x_{1},x_{2},\ldots ,x_{n}\right]}^{T}$ and $Y={\left[y_{1},y_{2},\ldots ,y_{n}\right]}^{T}$,

2.7 $$\sum_{i=1}^{n}\left|x_{i}\cdot y_{i}\right|\leq {\left(\sum_{i=1}^{n}{\left|x_{i}\right|}^{p}\right)}^{1/p}\cdot {\left(\sum_{i=1}^{n}{\left|y_{i}\right|}^{q}\right)}^{1/q},$$

where (1/p)+(1/q)=1 and p,q>1.

Based on the Holder inequality, for two discrete signal sequences $\left\{\;f_{1}(i)\geq 0,i=1,2,\ldots ,n\right\}$ and $\left\{\;f_{2}(i)\geq 0,i=1,2,\ldots ,n\right\}$, if (1/p)+(1/q)=1 and p,q>1, then the Holder coefficient of these two discrete signal sequences can be obtained as follows:

2.8 $$H_{c}=\frac{\sum_{i=1}^{n}\;f_{1}(i)\;f_{2}(i)}{{\left(\sum_{i=1}^{n}f_{1}^{p}(i)\right)}^{1/p}\cdot {\left(\sum_{i=1}^{n}f_{2}^{q}(i)\right)}^{1/q}},$$

where $0\leq H_{c}\leq 1$.

The Holder coefficient characterizes the degree of similarity of the two discrete signal sequences, and if and only if $f_{1}^{p}(i)=kf_{2}^{q}(i)$, i=1,2,…,n, in which *n* denotes the length of the discrete signal sequence and *k* is a real number, $H_{c}$ will be the maximum value. In this case, the degree of similarity of the two signal sequences is at its maximum, which indicates that these two signal sequences belong to the same type. If and only if $\sum_{i=1}^{n}\;f_{1}(i)\;f_{2}(i)=0$, $H_{c}$ will be the minimum value, and, in this case, the degree of similarity of the two signal sequences is at its minimum, which indicates that these two signal sequences are irrelevant and belong to different types.

The rectangular signal sequence $s_{1}(i)$ and the triangular signal sequence $s_{2}(i)$ are selected as a reference sequence. The Holder coefficient values between the bearing vibration signal sequence *f*(*i*) and the two reference signal sequences can be calculated, respectively.

The Holder coefficient value $H_{1}$ between the bearing vibration signal sequence *f*(*i*) and the rectangular signal sequence$s_{1}(i)$ can be obtained as follows:

2.9 $$H_{1}=\frac{\sum_{i=1}^{n}\;f(i)s_{1}(i)}{{\left(\sum_{i=1}^{n}\;f^{p}(i)\right)}^{1/p}\cdot {\left(\sum_{i=1}^{n}{s_{1}}^{q}(i)\right)}^{1/q}},$$

where the rectangular signal sequence $s_{1}(i)$ is expressed as

2.10 $$s_{1}(i)=\left\{ \begin{array}{l} 1,\;1\leq i\leq n,\\ 0,\;\text{else}. \end{array}\right.$$

In the same way, the Holder coefficient value $H_{2}$ between the vibration signal sequence *f*(*i*) and the triangular signal sequence $s_{2}(i)$,

2.11 $$H_{2}=\frac{\sum_{i=1}^{n}\;f(i)s_{2}(i)}{{\left(\sum_{i=1}^{n}\;f^{p}(i)\right)}^{1/p}\cdot {\left(\sum_{i=1}^{n}{s_{2}}^{q}(i)\right)}^{1/q}},$$

where the triangular signal sequence $s_{2}(i)$ is expressed as

2.12 $$s_{2}(i)=\left\{ \begin{array}{ll} 2i/n, & 1\leq i\leq n/2,\\ 2-2i/n, & n/2\leq i\leq n. \end{array}\right.$$

The Holder coefficient characteristics $\left[H_{1},H_{2}\right]$ are taken as another set of dominant feature vectors used for further rolling element bearing fault pattern recognition.

#### 2.1.3. Improved fractal box-counting dimension characteristics

Fractal theory is one of the most important branches of contemporary nonlinear sciences, and is suitable for processing all types of nonlinear and non-stationary phenomena and may also be suitable for fault feature extraction from bearing vibration signals. The fractal box-counting dimension algorithm has the advantage of being a simple calculation compared with other fractal dimension algorithms. The conventional algorithm of the fractal box-counting dimension has been widely used in the fields of image analysis, electromagnetic fault diagnosis and biomedicine, in which signals are strictly self-similar.

Suppose *A* is a non-empty bounded subset of Euclidean space *R^n^* to be calculated, and N(A,ε) is the least number of boxes with the side length ε covering *A*. Then the fractal box-counting dimension can be defined as

2.13 $$D=\underset{\varepsilon \to 0}{\lim}\frac{\log N(A,\varepsilon )}{\log(1/\varepsilon )}.$$

For the actual sampled vibration signal sequence f(i), $i=1,2,\ldots ,N_{0}$, there is no meaning for ε→0 to calculate the fractal box-counting dimension as the sampling interval σ is the highest resolution for the signal due to the existence of the sampling frequency. The minimum side length ε of the box is often made to be equal to σ. Consider the actual sampled bearing vibration signal sequence *f*(*i*) to be the closed set of Euclidean space *R^n^*, and the calculation process of the fractal box-counting dimension is described as follows.

Use the approximate method to make the minimum side length ε of the box covering the vibration discrete signal sequence *f*(*i*) equal to the sampling interval σ. Then calculate the least number of boxes N(kε) with side length kε covering the signal sequence *f*(*i*), thus:

2.14 $$\begin{array}{rl} p_{1} & =\max\{\;f(k(i-1)+1),f(k(i-1)+2),\ldots ,f(k(i-1)+k+1)\}, \end{array}$$

2.15 $$\begin{array}{rl} p_{2} & =\min\{\;f(k(i-1)+1),f(k(i-1)+2),\ldots ,f(k(i-1)+k+1)\} \end{array}$$

2.16 $$\begin{array}{rl} \text{and}\;p(k\varepsilon ) & =\sum_{i=1}^{N_{0}/k}\left|p_{1}-p_{2}\right|, \end{array}$$

where $i=1,2,\ldots ,N_{0}/k$, k=1,2⋯K. *N*_0_ is the number of sampling points, $K<N_{0}$. p(kε) is the longitudinal coordinate scale of the actual sampled bearing vibration signal sequence *f*(*i*). Thus $N_{k\varepsilon }$ can be defined as

2.17 N(kε)=p(kε)/kε+1.

Select a fitting curve log⁡kε∼log⁡N(kε) with good linearity as a scale-free zone, and the fitting curve can be defined as

2.18 log⁡N(kε)=alog⁡kε+b,

where $k_{1}\leq k\leq k_{2}$ and *k*_1_ and *k*_2_ are the start and end of the scale-free zone, respectively.

Generally, a least squares method is used to calculate the slope of the fitting curve, which is the fractal box-counting dimension *D* of the actual sampled bearing vibration signal sequence *f*(*i*),

2.19 $$D=-\frac{(k_{2}-k_{1}+1)\sum \log k\cdot \log N(k\varepsilon )-\sum \log k\cdot \sum \log N(k\varepsilon )}{(k_{2}-k_{1}+1)\sum \log^{2}k-{\left(\sum \log k\right)}^{2}}.$$

However, for the actual bearing vibration signals, they do not satisfy the self-similar structure of fractal theory to some degree. Therefore, when using the traditional fractal box-counting dimension algorithm to calculate the box-counting dimension of the vibration signals, the fitting curve often does not have a good linear structure. With the aim of resolving this issue, an improved generalized fractal box-counting dimension algorithm was developed to overcome the defect in the conventional fractal box-counting dimension algorithm. The specific calculation procedure is as follows.

(1) Resample the actual bearing vibration signal sequence *f*(*i*), $i=1,2,\ldots ,N_{0}$, and properly increase the sampling points to reduce the minimum side length ε, to improve the calculation accuracy of the fractal box-counting dimension of the signal sequence *f*(*i*). The phase space of the signal sequence *f*(*i*) is reconstructed, and the number of iterated dimensions of the reconstructed phase space is determined according to the number of sampling points.
(2) Suppose the number of sampling points of the signal sequence *f*(*i*) is $N_{0}=2^{n}$. To improve the calculation accuracy, resample the actual bearing vibration signal sequence *f*(*i*), and suppose that the number of sampling points of the signal sequence *f*(*i*) is $N=2^{K}$ (*K* > *n*). The reconstruction dimension of the phase space of the signal sequence *f*(*i*) is set, respectively, as $m=K+1=2,3,4,\ldots ,\log_{2}N+1$.
(3) The process of derivation of the number of boxes covering the actual bearing vibration signal sequence *f*(*i*) can be described as follows.
  When *k* = 1:
  $p_{1}=\max\{\;f(i),f(i+1)\}$, $p_{2}=\min\{\;f(i),f(i+1)\}$, i=1,2,…,N/k. In this case, the reconstructed phase space dimension is 2.
  When *k* = 2:
  $p_{1}=\max\{\;f(2i-1),f(2i),f(2i+1)\}$, $p_{2}=\min\{\;f(2i-1),f(2i),f(2i+1)\}$, i=1,2,…,N/k. In this case, the reconstructed phase space dimension is 3.
  When *k* = 3:
  $p_{1}=\max\{\;f(3i-2),f(3i-1),f(3i),f(3i+1)\}$, $p_{2}=\max\{\;f(3i-2),f(3i-1),f(3i),f(3i+1)\}$, i=1,2,…,N/k. In this case, the reconstructed phase space dimension is 4.
  When k=K:
  $p_{1}=\max\{\;f(Ki-K+1),f(Ki-K+2),\ldots ,f(Ki+1)\}$, $p_{2}=\min\{\;f(Ki-K+1),f(Ki-K+2),\ldots ,f(Ki+1)\}$, i=1,2,…,N/k. In this case, the reconstructed phase space dimension is *m* = *K* + 1.
(4) It can be seen from the above deduction that, during reconstructing the phase space of the bearing vibration signal sequence *f*(*i*) *K* times, the corresponding $\log N_{k\varepsilon }$ can be obtained at each time. Then the relation curve of $\log N_{k\varepsilon }∼\log k\varepsilon$ can be drawn. Since the fitting curve does not have a strict linear relationship, take the derivation of the relation curve at these *K* points over the improved generalized fractal box-counting dimension algorithm. The slopes $D_{1},D_{2},D_{3}\cdots D_{K}$ at these *K* points from the relation curve are the fractal box-counting dimensions in the different reconstructed phase space. Take the slopes $D_{1},D_{2},D_{3}\cdots D_{K}$ obtained as the *K* characteristic parameters for the fault feature vector extracted from the signal sequence *f*(*i*), which characterizes the bearing fault symptoms.

### 2.2. Grey relation pattern recognition

The study of grey relation theory is the foundation of grey system theory, which is mainly based on the basic theory of space mathematics, to calculate the relation coefficient and relation degree between the reference characteristic vector and each comparative characteristic vector. Grey relation theory has a good potential to be used in rolling element bearing fault classification for four reasons [32]: (i) it has good tolerance to measurement noise; (ii) its algorithm is simple and can solve the issue of generality versus accuracy; (iii) it can solve the learning problem with a small number of samples; and (iv) it has the ability to assist the selection of characteristic parameters for classification.

Suppose the health status feature vectors $\left\{E_{1},E_{2},H_{1},H_{2},D_{1},D_{2},D_{3},\ldots ,D_{K}\right\}$ (i.e. the multi-dimensional feature vectors extracted based on entropy characteristics, Holder coefficient characteristics and improved generalized fractal box-counting dimension characteristics) extracted based on vibration signals to be identified are as follows:

2.20 $$B_{1}=\left[\begin{array}{c} b_{1}(1)\\ b_{1}(2)\\ b_{1}(3)\\ \ldots \\ b_{1}(K+4) \end{array}\right],B_{2}=\left[\begin{array}{c} b_{2}(1)\\ b_{2}(2)\\ b_{2}(3)\\ \ldots \\ b_{2}(K+4) \end{array}\right],\ldots ,B_{i}=\left[\begin{array}{c} b_{i}(1)\\ b_{i}(2)\\ b_{i}(3)\\ \ldots \\ b_{i}(K+4) \end{array}\right],$$

where *B_i_* (\,*i* = 1,2,…) is a certain fault pattern to be recognized (i.e. fault types and severities).

Assume that the knowledge base between the health status patterns (i.e. fault types and severities) and the fault signatures (i.e. the health status feature vectors) based on some of the samples is as follows:

2.21 $$C_{1}=\left[\begin{array}{c} c_{1}(1)\\ c_{1}(2)\\ c_{1}(3)\\ \ldots \\ c_{1}(K+4) \end{array}\right],C_{2}=\left[\begin{array}{c} c_{2}(1)\\ c_{2}(2)\\ c_{2}(3)\\ \ldots \\ c_{2}(K+4) \end{array}\right],\ldots ,C_{j}=\left[\begin{array}{c} c_{j}(1)\\ c_{j}(2)\\ c_{j}(3)\\ \ldots \\ c_{j}(K+4) \end{array}\right],$$

where *C_j_* (*j* = 1,2,…) is a known health status pattern (i.e. fault types and severities) and *c_j_* (*j* = 1,2,…) is a certain feature parameter.

For ρ∈(0,1):

2.22 $$\xi (b_{i}(k),c_{j}(k))=\frac{\min_{j}\min_{k}|b_{i}(k)-c_{j}(k)|+\rho \cdot \max_{j}\max_{k}|b_{i}(k)-c_{j}(k)|}{\left|b_{i}(k)-c_{j}(k)|+\rho \cdot \max_{j}\max_{k}|b_{i}(k)-c_{j}(k)\right|}$$

and

2.23 $$\xi (B_{i},C_{j})=\frac{1}{K+4}\sum_{k=1}^{K+4}\xi (b_{i}(k),c_{j}(k)),\;j=1,2,\ldots ,$$

where ρ is the distinguishing coefficient; $\xi (b_{i}(k),c_{j}(k))$ is the grey relation coefficient of the *k*_th_ feature parameter for *B_i_* and *C_j_*;$\xi (B_{i},C_{j})$ is the grey relation degree for *B_i_* and *C_j_*. Thereafter *B_i_* is categorized to a certain fault pattern where the maximal $\xi (B_{i},C_{j})$ (*j* = 1,2,…,) is calculated.

### 2.3. Diagnostic procedure

**Figure 1.**
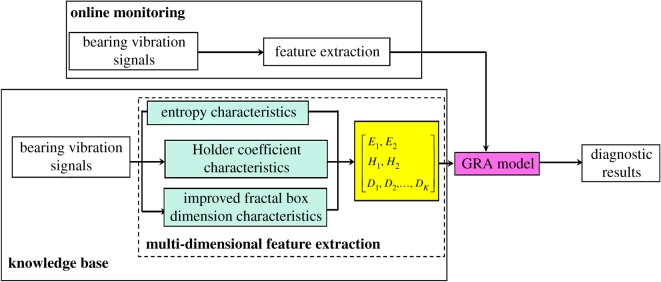
Rolling bearing online fault diagnosis based on multi-dimensional feature extraction.

In summary, the process of the proposed method for rolling bearing online fault diagnosis is as follows, and the flow chart is illustrated in figure 1.

Step 1: The vibration signals from the object bearing are sampled under different fault patterns, including normal operating conditions and conditions with different fault types and severities, to establish the knowledge base.

Step 2: The health status feature vectors are extracted from the sample knowledge base through the multi-dimensional feature extraction algorithm based on entropy characteristics, Holder coefficient characteristics and improved generalized fractal box-counting dimension characteristics.

Step 3: The sample knowledge base for the grey relation algorithm (GRA) is established based on the fault symptoms (i.e. the extracted fault feature vectors $\left\{E_{1},E_{2},H_{1},H_{2},D_{1},D_{2},D_{3},\ldots ,D_{K}\right\}$) and the fault pattern (i.e. the known fault types and severities).

Step 4: The health status feature vectors extracted based on bearing vibration signals to be identified are input into the GRA, and the diagnostic results (i.e. fault types and as well as severities) are output.

## 3. Experimental validation

**Figure 2.**
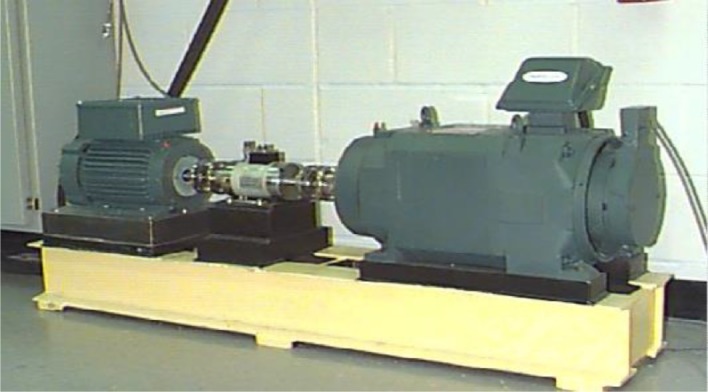
Experimental set-up.

In this paper, the rolling bearing vibration signals for testing are from the Case Western Reserve University Bearing Data Center [33]. The related rolling element bearing experimental device consists of a torque meter, a power meter and a three-phase induction motor, and the load power and speed are measured over the sensor as shown in figure 2. The motor drive end rotor is supported by a test bearing, where a single point of failure is set through discharge machining. The test bearing is a deep groove rolling bearing (6205–2RS JEM SKF). By controlling the power meter, the desired torque load can be obtained. The fault types consisted of an outer race fault, an inner race fault and a ball fault, and the fault diameters, i.e. fault severities, were 28 mils (1 mil = 0.0254 mm), 21 mils, 14 mils and 7 mils. An accelerometer was installed on the motor drive end housing with a bandwidth of up to 5000 Hz, and the vibration data for the test bearing under different fault patterns were collected by a recorder, in which the sampling frequency was 12 kHz.

The bearing vibration data used for analysis were obtained with a motor speed of 1797 rpm and load of 0 horsepower. In total, 11 types of vibration signals with different fault categories and severities were analysed, as seen in table 1. Each data sample from the vibration signals was made up of 2048 time-series points. For the 550 data samples, 110 data samples were chosen randomly to establish the knowledge base, with the remaining 440 data samples taken as the testing data samples. If the motor speed and the load changed in the practical application, data samples under these working conditions were taken to establish a new knowledge base so that the motor speed and load would not affect the diagnostic performance.

**Table 1.** Description of the experimental data set.

health status condition	fault diameter (mils)	the number of base samples	the number of testing samples	label of classification
normal	0	10	40	1
inner race fault	7	10	40	2
	14	10	40	3
	21	10	40	4
	28	10	40	5
ball fault	7	10	40	6
	14	10	40	7
	28	10	40	8
outer race fault	7	10	40	9
	14	10	40	10
	21	10	40	11

The signal feature vectors extracted by entropy (Shannon entropy and exponential entropy) and Holder coefficient theories in [29,30,34] achieved good performance. Also, the calculation process of the entropy characteristics and the Holder coefficient characteristics is simple, which means the generality and the accuracy of the feature extraction algorithm can be easily balanced. In addition, in order to improve the rolling bearing fault diagnostic performance to meet the requirements of accurate diagnosis of different fault types and severities with real-time computational performance, we proposed a multi-dimensional feature extraction algorithm on the basis of the entropy characteristics, the Holder coefficient characteristics and the improved generalized fractal box-counting dimension characteristics.

**Figure 3.**
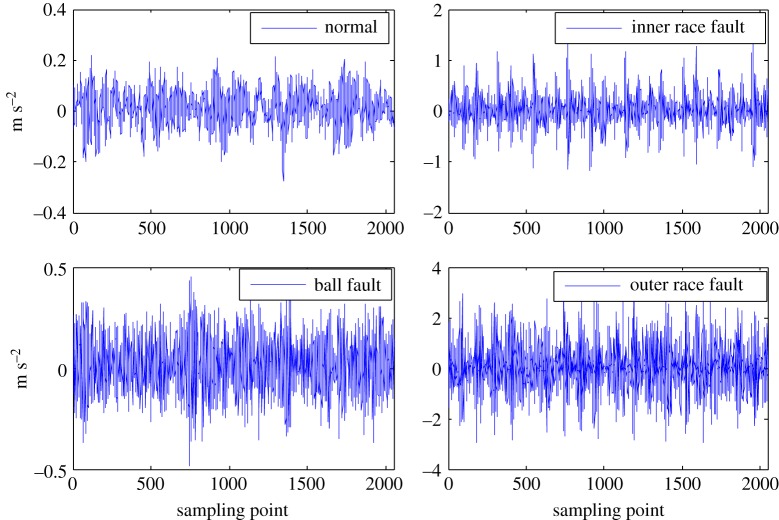
Rolling bearing normal operating condition and various fault conditions with fault diameter 7 mils.

**Figure 4.**
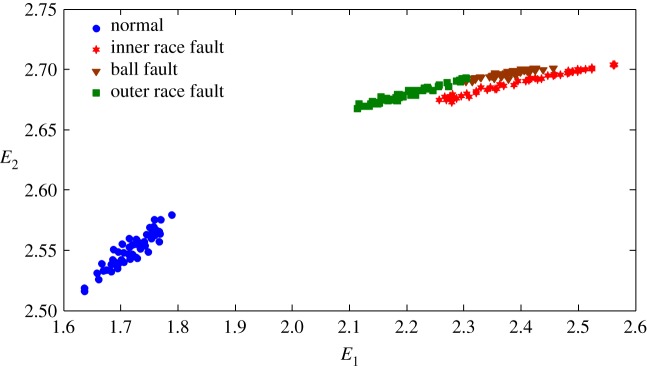
Entropy features of a randomly selected sample from normal operating conditions and various fault conditions with fault diameter 7 mils, where the *x*-axis *E*_1_ represents the Shannon entropy, and the *y*-axis *E*_2_ represents the exponential entropy.

**Figure 5.**
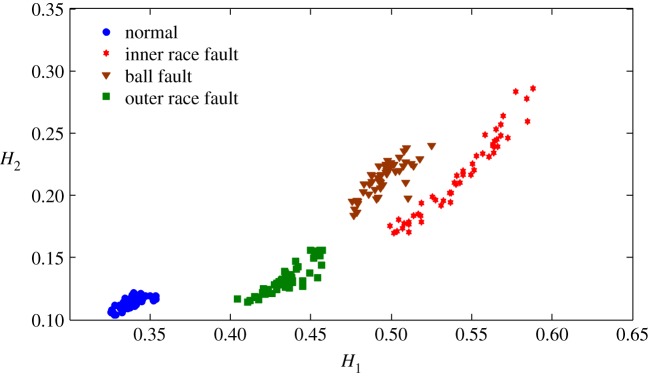
Holder coefficient features of a randomly selected sample from normal operating conditions and various fault conditions with fault diameter 7 mils, where the *x*-axis *H*_1_ represents the Holder coefficient with the rectangular sequence as the reference sequence, and the *y*-axis *H*_2_ represents the Holder coefficient with the triangular sequence as the reference sequence.

**Figure 6.**
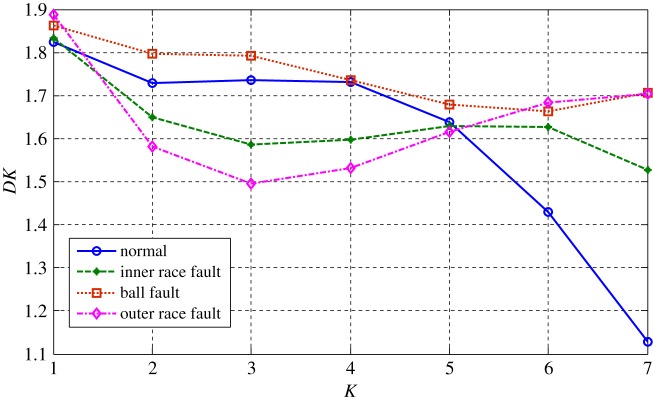
Improved generalized fractal box-counting dimension of a randomly chosen sample from bearing normal conditions and different fault conditions with fault size 7 mils.

**Figure 7.**
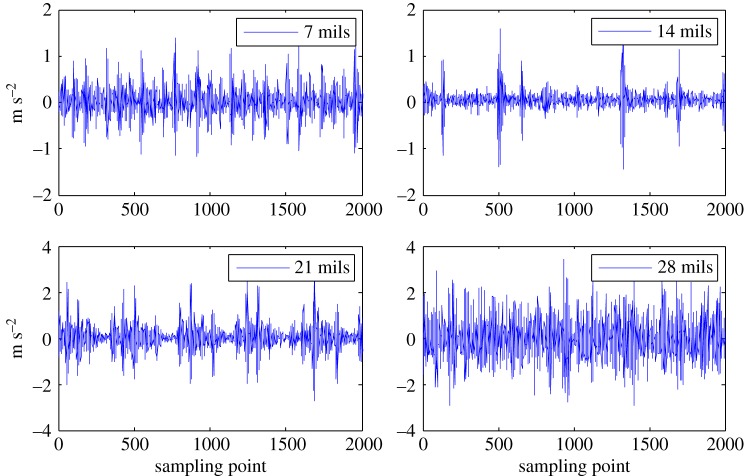
Bearing inner race fault conditions with various severities.

**Figure 8.**
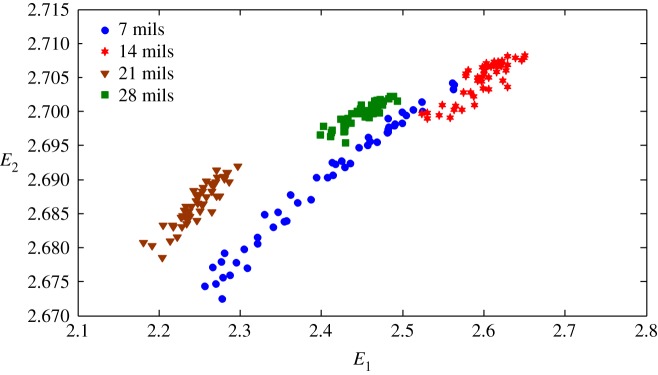
Entropy features of a randomly selected sample from the inner race fault condition with various severities.

**Figure 9.**
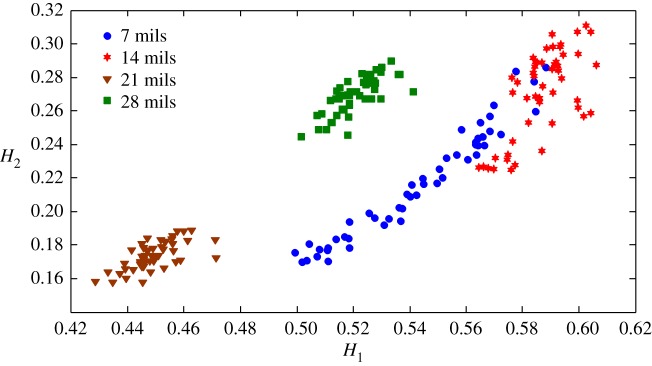
Holder coefficient features of a randomly selected sample from the inner race fault condition with various severities.

**Figure 10.**
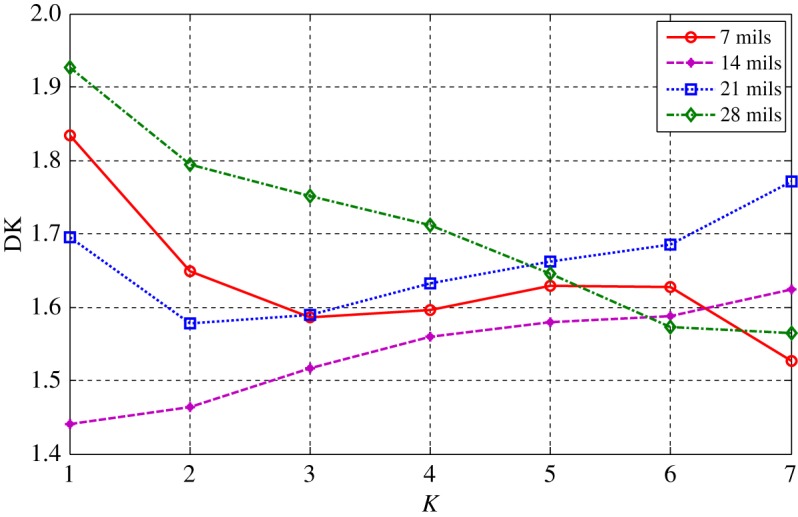
Improved generalized box-counting dimension of a randomly chosen sample from the bearing inner race fault condition with different levels of severity.

The health status feature vectors extracted from the rolling bearing normal operating conditions and different fault conditions with 7 mils fault diameter (seen in figure 3) based on the entropy characteristics, Holder coefficient characteristics and improved generalized fractal box-counting dimension characteristics are shown in figures 4–6, respectively. Also, the health status feature vectors extracted from the inner race fault condition with various severities (seen in figure 7) based on the entropy characteristics, Holder coefficient characteristics and improved generalized box-counting dimension characteristics are shown in figures 8–10, respectively.

From figures 4–6, it can be seen that both the Holder coefficient characteristics and the improved generalized fractal box-counting dimension characteristics of a randomly chosen sample from bearing normal conditions and different fault conditions with 7 mils fault diameter show clear differences, while the entropy characteristics of a randomly chosen sample from different fault conditions with the same fault size are very similar.

From figures 8–10, it can be seen that the improved generalized fractal box-counting dimension characteristics of a randomly chosen sample from the bearing inner race fault condition with different levels of severity show clear differences, while both the entropy characteristics and the Holder coefficient characteristics show some overlapping features between the randomly chosen sample from the bearing inner race fault condition with 7 mils fault diameter and the randomly chosen sample from the bearing inner race fault condition with 14 mils fault diameter.

After the dominant fault feature vectors $\left\{E_{1},E_{2},H_{1},H_{2},D_{1},D_{2},D_{3},\ldots ,D_{K}\right\}$ were extracted from the rolling element bearing vibration signals with different fault types and severities through the multi-dimensional feature extraction algorithm based on the entropy characteristics, the Holder coefficient characteristics and improved generalized fractal box-counting dimension characteristics, the sample knowledge base for the GRA was established based on the fault symptoms (i.e. the extracted health status feature vectors $\left\{E_{1},E_{2},H_{1},H_{2},D_{1},D_{2},D_{3},\ldots ,D_{K}\right\}$) and the fault pattern (i.e. the known fault types and severities). The fault feature vectors $\left\{E_{1},E_{2},H_{1},H_{2},D_{1},D_{2},D_{3},\ldots ,D_{K}\right\}$ extracted from the test rolling bearing vibration signals to be identified were input to the GRA, and the diagnostic results (i.e. fault types and severities) were output, as shown in table 2.

**Table 2.** The diagnostic results by the proposed method compared with results from [34–36]. Note: The approach of [35] is based on multifractal theory for extracting feature vectors and a GRA for achieving pattern recognition intelligently using the extracted feature vectors. The approach of [36] is based on the improved generalized fractal box-counting dimension and adaptive GRA for achieving pattern recognition intelligently using the extracted feature vectors. The approach of [34] is based on a four-dimensional feature extraction algorithm using the entropy and Holder coefficient theories for extracting feature vectors and a GRA for achieving pattern recognition intelligently using the extracted feature vectors. In these previous works, such as [34–36], the authors have fully compared the recognition results with the existing feature extraction algorithm (such as the entropy theory, the Holder coefficient theory and multifractal theory) and pattern recognition algorithm (such as the feed-forward back-propagation neural network and the SVM, the adaptive GRA) in the same topic. At this stage, we propose the improved algorithm based on these previous works [34–36] to improve the rolling bearing signal subtle feature extraction performance.

		number of misclassified samples				testing accuracy (%)			
label of classification	number of test samples	[35]	[36]	[34]	proposed	[35]	[36]	[34]	proposed
1	40	0	0	0	0	100	100	100	100
2	40	0	0	0	0	100	100	100	100
3	40	0	4	2	1	100	90	95	97.5
4	40	3	0	0	0	92.5	100	100	100
5	40	0	0	0	0	100	100	100	100
6	40	2	4	3	2	95	90	92.5	95
7	40	3	0	0	3	92.5	100	100	92.5
8	40	3	4	4	0	92.5	90	90	100
9	40	0	0	0	0	100	100	100	100
10	40	0	0	3	0	100	100	92.5	100
11	40	4	4	0	1	90	90	100	97.5
in total	440	15	16	12	7	96.59	96.3636	96.9697	98.4091

The diagnostic results from table 2 illustrate that the diagnostic success rate for a faulty bearing can reach 100%, with a total diagnostic success rate of almost 98.4%, which shows a definite improvement in the diagnostic accuracy after the application of the improved fractal box-counting dimension in rolling bearing fault diagnosis based on multi-dimensional feature extraction, compared with the methods from [34–36]. The time cost of the methods on a laptop computer with a 4.0 GHz dual processor for one test case is only 0.013 s. The time consumption of the proposed approach is encouraging, and the proposed approach is very suitable for online health status evaluation.

## 4. Conclusion

In this paper, an effective rolling bearing fault diagnostic method was developed to meet the requirements for accurate diagnosis of different fault types and severities with real-time computational performance after integration of the improved fractal box-counting dimension into the multi-dimensional characteristics. The experimental study has illustrated the following.

(1) The proposed method can accurately and effectively recognize different types of rolling bearing failure and the severities of the fault.
(2) The diagnostic results by the proposed method show that the diagnostic success rate for bearing faulty conditions can reach 100%, with a total diagnostic success rate of almost 98.4%.
(3) The proposed method can improve the fault diagnostic performance compared with the existing pattern recognition methods, and is very suitable for online health status assessment.

In future research, based on the real-time performance algorithm, in order to continually improve fault diagnostic accuracy, this study on rolling bearing online fault diagnosis will be extended with the integration of multi-dimensional feature extraction and Dempster–Shafer evidence theory.

## Data accessibility

The rolling bearing vibration signals for testing were from the Case Western Reserve University Bearing Data Center [33].

## References

[1] Li J, Ying Y 2018 A method to improve the robustness of gas turbine gas-path fault diagnosis against sensor faults. IEEE Trans. Reliability 67, 3–12. (doi:10.1109/TR.2017.2695482)

[2] Jiang L, Shi T, Xuan J 2012 Fault diagnosis of rolling bearings based on marginal fisher analysis. J. Vibr. Control 20, 470–480. (doi:10.1177/1077546312463747)

[3] Van Hecke B, Qu Y, He D 2015 Bearing fault diagnosis based on a new acoustic emission sensor technique. Proc. Inst. Mech. Eng. [O] 229, 105–118. (doi:10.1177/1748006X14558900)

[4] Xu J et al. 2015 The application of time–frequency reconstruction and correlation matching for rolling bearing fault diagnosis. Proc. Inst. Mech. Eng. [C] 229, 3291–3295. (doi:10.1177/0954406215584397)

[5] Zhang X et al. 2015 A bearing fault diagnosis method based on the low-dimensional compressed vibration signal. Adv. Mech. Eng. 7, 1687814015593442.

[6] Yun L, Can W, Chunguang M, Zheng D, Xuefei M 2016 A new combination method for multisensor conflict information. J. Supercomput, 1–17. (doi:10.1007/s11227-016-1681-3)

[7] Zhang DD 2011 Bearing fault diagnosis based on the dimension–temporal information. Proc. Inst. Mech. Eng. [J] 225, 806–813. (doi:10.1177/1350650111410254)

[8] Vakharia V, Gupta VK, Kankar PK 2014 A multiscale permutation entropy based approach to select wavelet for fault diagnosis of ball bearings. J. Vibr. Control 17, 201812094.

[9] Tiwari R, Gupta VK, Kankar PK 2015 Bearing fault diagnosis based on multi-scale permutation entropy and adaptive neuro fuzzy classifier. J. Vibr. Control 21, 461–467. (doi:10.1177/1077546313490778)

[10] Sun W et al. 2013 Fault diagnosis of rolling bearing based on wavelet transform and envelope spectrum correlation. J. Vibr. Control 19, 924–941. (doi:10.1177/1077546311435348)

[11] Wang H, Chen J, Dong G 2014 Fault diagnosis of rolling bearing's early weak fault based on minimum entropy de-convolution and fast Kurtogram algorithm. Proc. Inst. Mech. Eng. [C] 229, 2890–2907. (doi:10.1177/0954406214564692)

[12] Yunusa-Kaltungo A, Sinha JK 2014 Faults diagnosis in rotating machines using higher order spectra. In *Proc. of ASME Turbo Expo 2014: Turbine Technical Conference and Exposition*. New York, NY: American Society of Mechanical Engineers.

[13] Liu Q, Chen F, Zhou Z, Wei Q 2013 Fault diagnosis of rolling bearing based on wavelet package transform and ensemble empirical mode decomposition. Adv. Mech. Eng. 5, 792584 (doi:10.1155/2013/792584)

[14] Cai J 2014 Fault diagnosis of rolling bearing based on empirical mode decomposition and higher order statistics. Proc. Inst. Mech. Eng. [C] 229, 1630–1638. (doi:10.1177/0954406214545820)

[15] Lin Y, Wang C, Wang J, Dou Z 2016 A novel dynamic spectrum access framework based on reinforcement learning for cognitive radio sensor networks. Sensors 16, 1–22. (doi:10.1109/JSEN.2016.2616227) 10.3390/s16101675 PMC5087463 27754316

[16] Zhu K, Li H 2015 A rolling element bearing fault diagnosis approach based on hierarchical fuzzy entropy and support vector machine. Proc. Inst. Mech. Eng. [C] 230, 2314–2322. (doi:10.1177/0954406215593568)

[17] Zhu K, Song X, Xue D 2014 A roller bearing fault diagnosis method based on hierarchical entropy and support vector machine with particle swarm optimization algorithm. Measurement 47, 669–675. (doi:10.1016/j.measurement.2013.09.019)

[18] Zheng J, Cheng J, Yang Y 2013 A rolling bearing fault diagnosis approach based on LCD and fuzzy entropy. Mech. Machine Theory 70, 441–453. (doi:10.1016/j.mechmachtheory.2013.08.014)

[19] Xiong G, Zhang L, Liu H, Guo W-Z 2010 A comparative study on ApEn, SampEn and their fuzzy counterparts in a multiscale framework for feature extraction. J. Zhejiang Univ. Sci. A 11, 270–279. (doi:10.1631/jzus.A0900360)

[20] Yan R, Gao RX 2004 Machine health diagnosis based on approximate entropy. In *Proc. 21st IEEE Instrumentation and Measurement Technology Conference (IMTC 04), Como, Italy, 18–20 May 2004*, vol. 3, pp. 2054–2059. New York, NY: IEEE.

[21] Yan R, Gao RX 2007 Approximate entropy as a diagnostic tool for machine health monitoring. Mech. Syst. Signal Process. 21, 824–839. (doi:10.1016/j.ymssp.2006.02.009)

[22] Dong S, Xu X, Liu J, Gao Z 2015 Rotating machine fault diagnosis based on locality preserving projection and back propagation neural network–support vector machine model. Measure. Control 48, 211–216. (doi:10.1177/0020294015595995)

[23] Jayaswal P, Verma SN, Wadhwani AK 2011 Development of EBP-artificial neural network expert system for rolling element bearing fault diagnosis. J. Vibr. Control 17, 1131–1148. (doi:10.1177/1077546310361858)

[24] Wang CC, Kang Y, Shen PC, Chang Y-P, Chung Y-L 2010 Applications of fault diagnosis in rotating machinery by using time series analysis with neural network. Expert System Appl. 37, 1696–1702. (doi:10.1016/j.eswa.2009.06.089)

[25] Samanta B, Al-Balushi KR 2003 Artificial neural network based fault diagnostics of rolling element bearings using time-domain features. Mech. Syst. Signal Process. 17, 317–328. (doi:10.1006/mssp.2001.1462)

[26] Ao HL et al. 2013 The support vector machine parameter optimization method based on artificial chemical reaction optimization algorithm and its application to roller bearing fault diagnosis. J. Vibr. Control 21, 2434–2445. (doi:10.1177/1077546313511841)

[27] Zhang XL, Chen XF, He ZJ 2010 Fault diagnosis based on support vector machines with parameter optimization by an ant colony algorithm. Proc. Inst. Mech. Eng. [C] 224, 217–229. (doi:10.1243/09544062JMES1731)

[28] Hsu CW, Lin CJ 2002 A comparison of methods for multiclass support vector machines. IEEE Trans. Neural Netw. 13, 415–425. (doi:10.1109/72.991427) 18244442 10.1109/72.991427

[29] Li J, Guo J 2015 A new feature extraction algorithm based on entropy cloud characteristics of communication signals. Math. Problem. Eng. 2015 (doi:10.1155/2015/891731)

[30] Li J 2015 A new robust signal recognition approach based on Holder cloud features under varying SNR environment. KSII Trans. Internet Inform. Syst. 9, 4934–4949.

[31] Li J 2015 A novel recognition algorithm based on Holder coefficient theory and interval gray relation classifier. KSII Trans. Internet Inform. Syst. (TIIS) 9, 4573–4584.

[32] Ying Y, Cao Y, Li S, Li J, Guo J 2016 Study on gas turbine engine fault diagnostic approach with a hybrid of gray relation theory and gas-path analysis. Adv. Mech. Eng. 8, 1–14. (doi:10.1177/1687814015627769)

[33] The Case Western Reserve University Bearing Data Center. See http://csegroups.case.edu/bearingdatacenter/pages/download-data-file (accessed 11 October 2015).

[34] Ying Y, Li J, Chen Z, Guo J 2017 Study on rolling bearing online reliability analysis based on vibration information processing. Comput. Electric. Eng. (doi:10.1016/j.compeleceng.2017.11.029)

[35] Li J, Cao Y, Ying Y, Li S 2016 A rolling element bearing fault diagnosis approach based on multifractal theory and gray relation theory. PLoS ONE 11, 1–16. (doi:10.1371/journal.pone.0167587) 10.1371/journal.pone.0167587 PMC5201295 28036329

[36] Cao Y, Ying Y, Li J, Guo J 2016 Study on rolling bearing fault diagnosis approach based on improved generalized fractal box-counting dimension and adaptive gray relation algorithm. Adv. Mech. Eng. 8, 1–11. (doi:10.1177/1687814016675583)

